# Resistive Switching Devices for Neuromorphic Computing: From Foundations to Chip Level Innovations

**DOI:** 10.3390/nano14060527

**Published:** 2024-03-15

**Authors:** Kannan Udaya Mohanan

**Affiliations:** Department of Electronic Engineering, Gachon University, Seongnam-si 13120, Gyeonggi-do, Republic of Korea; kannan_um@gachon.ac.kr

**Keywords:** neuromorphic computing, resistive switching, neuromorphic chip, synapse, neuron, deep learning, memristor

## Abstract

Neuromorphic computing has emerged as an alternative computing paradigm to address the increasing computing needs for data-intensive applications. In this context, resistive random access memory (RRAM) devices have garnered immense interest among the neuromorphic research community due to their capability to emulate intricate neuronal behaviors. RRAM devices excel in terms of their compact size, fast switching capabilities, high ON/OFF ratio, and low energy consumption, among other advantages. This review focuses on the multifaceted aspects of RRAM devices and their application to brain-inspired computing. The review begins with a brief overview of the essential biological concepts that inspire the development of bio-mimetic computing architectures. It then discusses the various types of resistive switching behaviors observed in RRAM devices and the detailed physical mechanisms underlying their operation. Next, a comprehensive discussion on the diverse material choices adapted in recent literature has been carried out, with special emphasis on the benchmark results from recent research literature. Further, the review provides a holistic analysis of the emerging trends in neuromorphic applications, highlighting the state-of-the-art results utilizing RRAM devices. Commercial chip-level applications are given special emphasis in identifying some of the salient research results. Finally, the current challenges and future outlook of RRAM-based devices for neuromorphic research have been summarized. Thus, this review provides valuable understanding along with critical insights and up-to-date information on the latest findings from the field of resistive switching devices towards brain-inspired computing.

## 1. Introduction

In the modern technology landscape, advancements in diverse fields such as big data analytics, internet-of-things (IOT), deep learning, self-driving autonomotive technology, edge computing applications, etc. are accompanied by a pressing demand for higher computational power at a lower energy budget. Present-day computer systems are almost exclusively designed on the principle of von Neumann architecture, where the memory and central processing units are physically separate. Due to the requirement of constant data flow between these two units, conventional digital computers suffer from huge latency and energy expenditure. This is often referred to as the “von Neumann bottleneck”, which critically impedes the computing capabilities of these systems. The requirement for separate memory and processing units also reduces the future scaling of these devices for increasing data-centric applications. Neuromorphic computing, or bio-inspired computing [1], is a promising alternative where the computing design is derived from the workings of the human brain. A striking feature of the human brain is the large amount of parallelism achieved through a complex network of neurons (∼10^11^) and synapses (∼10^15^). These ultra-low-power computing elements (neurons and synapses) can perform much better than the current computing systems in applications like pattern recognition, data classification, etc. Synapses, which are the junction between adjacent neurons, form the basic component of learning and memory, which is achieved through the modulation of synaptic weights. Essentially, a biological synapse consists of the axon terminal of a pre-synapse, the dendrite terminal of the post-synapse, and a synaptic cleft in between these two terminals. Neurotransmitters are released between the pre- and post-synapses through the synaptic cleft based on the modulation of the synaptic weights. Synaptic plasticity is the property by which the synaptic weight changes based on the activity at its neurons. The human brain exhibits a wide range of synaptic plasticity schemes like short-term potentiation (STP), long-term potentiation (LTP), spike-timing-dependent plasticity (STDP), etc., which helps in memory retention and computing capabilities. It is indeed necessary for any neuromorphic system to faithfully emulate these plasticity responses of the human brain to achieve a similar level of system efficiency in learning and processing domains.

Hardware realization of such a complex neural network was initially attempted with the conventional complementary metal oxide semiconductor (CMOS) transistors. With immense research effort, CMOS-based neuromorphic chip designs like the TrueNorth [2], BrainScales [3], and Loihi 2 [4] have achieved break-through performance records. In 2023, IBM released the NorthPole chip [5], which is reported to be 25 times more energy efficient than corresponding graphical processing units (GPUs) in handling deep learning workloads. But the overall energy consumption and chip footprint still need to be considerably lowered. As an alternative, several device architectures, including three terminal devices like ferroelectric field effect transistors [6], electrochemical transistors [7], etc., have been attempted for neuromorphic applications. However, emerging non-volatile memory (eNVM) devices such as phase change memory (PCM) [8], magnetoresistive RAM (MRAM) [9], ferroelectric RAM (FeRAM) [10] and resistive switching RAM (RRAM) [11] are particularly attractive because of the promise of scalability and ease of fabrication. Unlike conventional charge-based devices like static RAM (SRAM), dynamic RAM (DRAM), and flash memory, these eNVM devices function based on the underlying physics of their constituent layers. Table 1 shows a summary of the important performance metrics among the eNVM devices. Overall, RRAM devices, with their minimal power consumption (∼0.1 pJ per write operation), rapid read time (∼1 ns), and compact size (∼4 F^2^, where F represents the minimum feature size of the technology node), present a compelling option for both non-volatile data storage and addressing current challenges in device scaling. They have the basic structure of an insulating material sandwiched between two metallic electrodes. They work on the basis of the formation and rupture of a conductive filament in the insulating layer between the two electrodes. A memristor is a special kind of RRAM device with a pinched hysteresis loop [12]. They have a continuous response to the variation of input voltage, which is identical to the response of biological systems to an external stimulus. Hence, they are ideally suited for handling the complexities of biological neural networks. RRAM devices are looked upon as integral components not only for neuromorphic computing design but also for addressing contemporary computational challenges through their enhanced processing efficiency and storage density.

**Figure 1:**
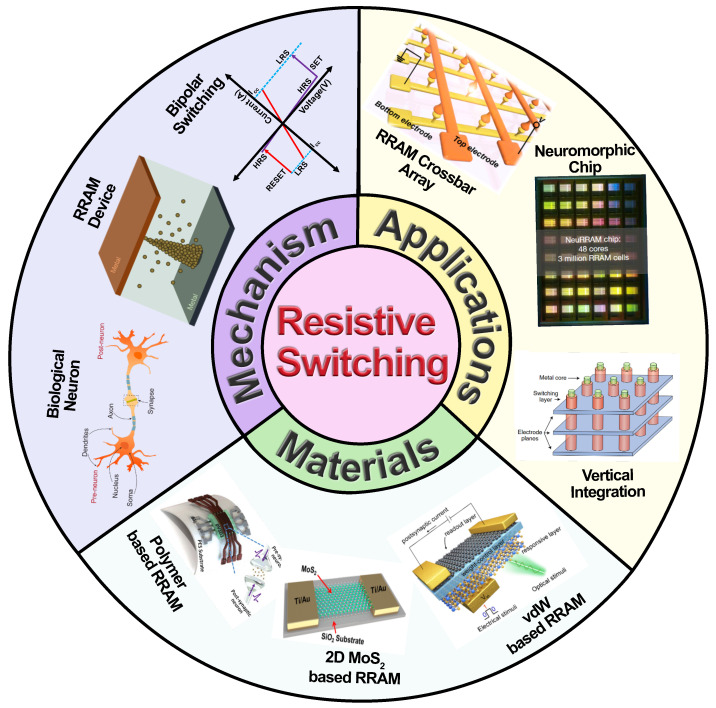
Schematic overview of the various topics covered in this review article. Inset images of figure reprinted with permission from [13,14,15,16,17,18]. Copyright © 2024 American Chemical Society. Copyright © 2024 Springer Nature.

In the current article, we present a comprehensive review of the basic concepts and various latest results in RRAM device research for neuromorphic applications. Figure 1 depicts a schematic overview of the various topics covered in this review article. First, the biological background pertinent to neuromorphic device research is discussed. This includes the basic organization of the neuron and its inter-neuron communication modulated through various synaptic plasticities like STDP, spike-rate-dependent plasticity (SRDP), etc. In addition, advanced synaptic behaviors like heteroplasticity and metaplasticity are also discussed. The physical realization of such synaptic functionalities requires a detailed understanding of resistive switching working mechanism. Hence, the review presents a detailed overview of the basic working principles of RRAM devices and their various types of switching mechanisms. In addition to well-known switching mechanisms like ion migration, oxygen vacancy-based, and trap assisted switching, other mechanisms like ion intercalation, ferroelectric switching, and spin orbit torque-based switching mechanisms would also be discussed. A wide variety of materials including oxides, chalcogenides, nitrides, organic polymers, and biomaterials, have been used for fabricating RRAM devices. Recently, lower-dimensional systems like 2D materials, quantum dots, and nanowires have also been used to mimic neuromorphic behaviors using RRAM devices. Herein, the review also elaborates on the various material choices and related experimental results for RRAM devices for neuromorphic applications. Next, a comprehensive overview of some of the emerging trends in RRAM based neuromorphic applications is discussed, with a special emphasis on some of the large-scale CMOS compatible chip-based implementations. Finally, the review summarizes the various challenges and future outlook for RRAM-based neuromorphic research.

**Table 1:** Comparison of eNVM devices [11,19] based on performance attributes.

Performance Metrics	RRAM	FeRAM	MRAM	PCM
Cell area	4 F^2^	22 F^2^	6–50 F^2^	4–20 F^2^
Write voltage (V)	<2	<3	<2	<3
Read time (ns)	∼1	<5	∼20	<10
Write energy	∼0.1 pJ	∼30 fJ	∼0.1 pJ	∼10 pJ
Retention (s)	>10^6^	>10^6^	>10^6^	>10^6^
Endurance (cycles)	>10^12^	>10^14^	>10^15^	$10^{6}$–$10^{9}$

## 2. Biological Background

**Figure 2:**
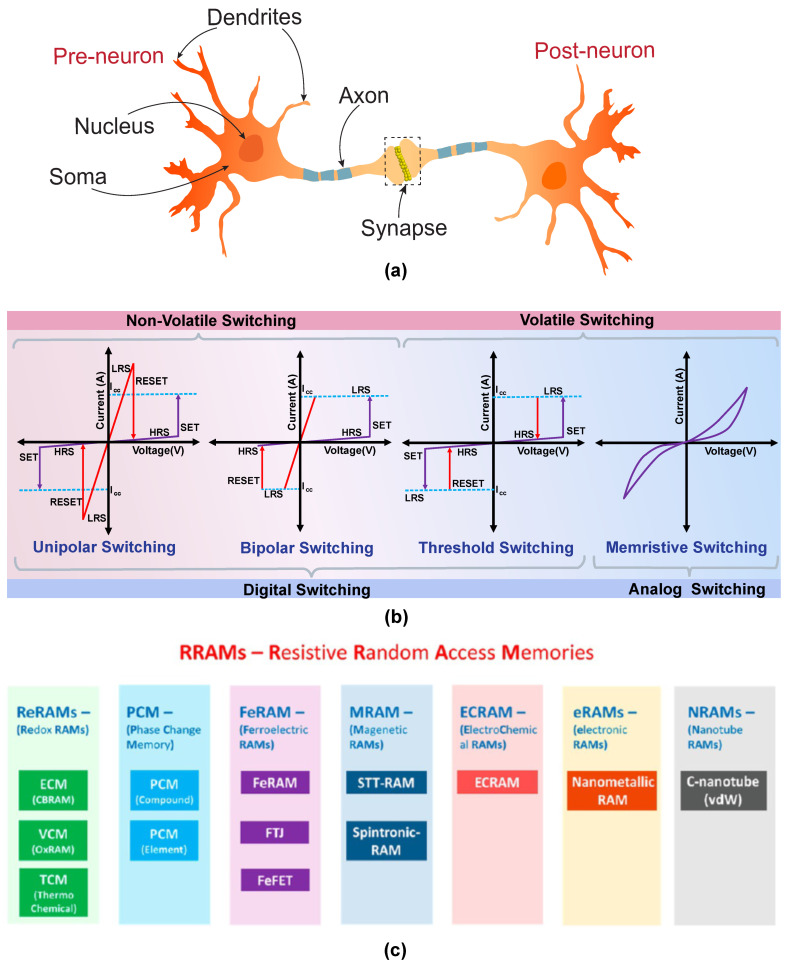
(**a**) Schematic of the major components of a biological neuron. (**b**) Illustration of the various types of switching mechanisms observed in RRAM devices. (**c**) Schematic description of the different RRAM device types based on their operation mechanism. Reprinted with permission from [24]. Copyright © 2024, American Chemical Society.

The primary components of the neural circuitry are the neurons (see Figure 2a), which receive signals through the “dendrites”, which are connected to adjacent neurons. These signals are processed at the “soma”, which plays the role of a ’central processing unit’ in a conventional computing architecture. The soma processes the received signals through a non-linear transformation and generates an output signal if the accumulated input crosses a threshold value. The generated output signal is then transmitted through the “axon”, which acts as the output device. The axon transfers the electrical signals to adjacent neurons through a narrow junction called the “synaptic junction” or the “synapse”. Such a synapse is typically formed between the axon terminal of one neuron (the preneuron) and the dendrite receptors of another (the postneuron [20,21]). The strength of such a synaptic connection determines the “synaptic weight”, which controls the efficiency of information transfer across adjacent neurons. The synaptic weights can be modulated using a property called the “synaptic plasticity” [22]. Synaptic plasticity is responsible for the learning and memory capabilities of the neural network. Depending upon the relative activities of the pre- and post-neurons, several types of plasticities have been discovered [23], which include potentiation & depression, spike-time-dependent plasticity (STDP), spike-rate-dependent plasticity (SRDP), metaplasticity, heteroplasticity, etc. The increase or decrease in the post-neuron activity as a result of the relative variation in preneuronal spike timing, rate, etc. is called potentiation/depression. A prerequisite for the potentiation or depression action is that the synaptic strength should be adjustable based on the external stimulus. Potentiation and depression are broadly classified into four types—short-term potentiation (STP), short-term depression (STD), long-term potentiation (LTP), and long-term depression (LTD). STP & STD are characterized by the temporary potentiation/depression of the synaptic weight, which lasts for only a few seconds or minutes and then decays to its initial value. STP/STD are particularly relevant for short-term information processing within the neural circuitry. LTP & LTD extend over several minutes, hours, or even days and are non-volatile in nature. LTP/LTD plays a significant role in the learning and memory activities of the brain. The Hebbian rule postulated in 1949 established that the synaptic connection strength between a pre- and post-neuron depends on the simultaneous activity of both neurons [22]. The rule forms the basis of the learning and memory capabilities of the neural network. STDP is a form of Hebbian learning that works based on the relative timing of the application of spikes. According to the rule, the change in synaptic weight Δw depends on the time difference Δt between pre- and post-spikes. If a preneuron spikes before a postneuron spike (Δt>0), the synaptic weight Δw increases or the synapse potentiates. Whereas if a preneuron spikes after a postneuron spike (Δt<0), Δw decreases or the synapse depresses.

SRDP is a form of synaptic plasticity where the synaptic weight change is determined by the frequency change of the spiking rate between the pre- and post-synapses. Metaplasticity is another kind of learning behavior that shows the effect of stimulus history on the synapse response. The activation of metaplasticity requires the application of a priming stimulus, which does not cause any major change in synaptic conductance. However, such a pre-stimulus spike causes a change in the behavior of synapses during further stimulus application. Heterosynaptic plasticity originates from the action of an extra interneuron, which modulates the synaptic plasticity behavior between the pre- and post-neurons. Unlike the homosynaptic responses discussed before, where the same set of neurons are used for sourcing the spikes and sensing the synaptic weight change, heterosynaptic plasticity depends on the influence of a third neuron, which acts as a modulatory terminal. Such a synaptic behavior is biologically significant for associative learning, sensory perception, long-term memory, etc. The various plasticity mechanisms discussed here provide definitive test cases that RRAM devices need to emulate effectively for their practical use in brain-inspired computing.

## 3. Resistive Switching

### 3.1. Types of Resistive Switching

Resistive switching phenomenon can be broadly classified into digital and analog switching based on the nature of current-voltage characteristics observed in the device.

Figure 2b shows a schematic description of the various types of switching mechanisms commonly observed in RRAM devices. Digital switching denotes the sudden jump in current flowing through the device at a particular voltage applied to the device, called the set voltage. Here, the switching process is sudden and abrupt resulting in sharply differentiated high-resistance (HRS) and low-resistance states (LRS). Digital switching is extremely critical for developing memory devices with a clear distinction between ON and OFF states. This type of switching is further divided into bipolar, unipolar, and threshold resistive switching. In bipolar resistive switching, the set and reset switching occur at the opposite polarity of applied voltages. Conversely, in unipolar switching, both the set and reset switches occur at the same polarity. Unipolar switching is independent of the polarity of the applied voltage, and it occurs mainly due to the Joule-heating-induced rupture of the conductive filaments formed inside the resistive switching medium. Both bipolar and unipolar resistive switching devices retain their memory states even after the applied voltage is removed, and hence they are referred to as non-volatile memory devices. Threshold switching is a type of volatile resistive switching where the device resets to the HRS state when the applied voltage drops below a certain threshold value. Hence, this type of resistive switching device cannot retain its LRS state once the applied voltage is removed. Analog resistive switching is the second type of switching where the change in resistance states is a gradual and continuous process. Such a type of continuous state change is similar to the responses observed in biological systems and, hence, is ideally suited for neuromorphic applications. Analog resistive switching is experimentally equivalent to the circuit element referred to as memristor [12], where the device has an inherent memory of its resistance state. RRAM devices exhibiting analog switching characteristics are often interchangeably referred to as memristors as well [25].

### 3.2. Resistive Switching Mechanisms

The resistive switching effect in memristors could arise from several possible mechanisms. Figure 2c shows a general schematic depicting the wide range of RRAM devices based on their physical mechanisms. Here, we discuss some of the prominent switching mechanisms like ion migration, trap assisted switching, ferroelectric & magnetic tunnel junction-based switching, metal-insulator transition based switching, ion intercalation, etc.

#### 3.2.1. Ion Migration Based Switching

**Figure 3:**
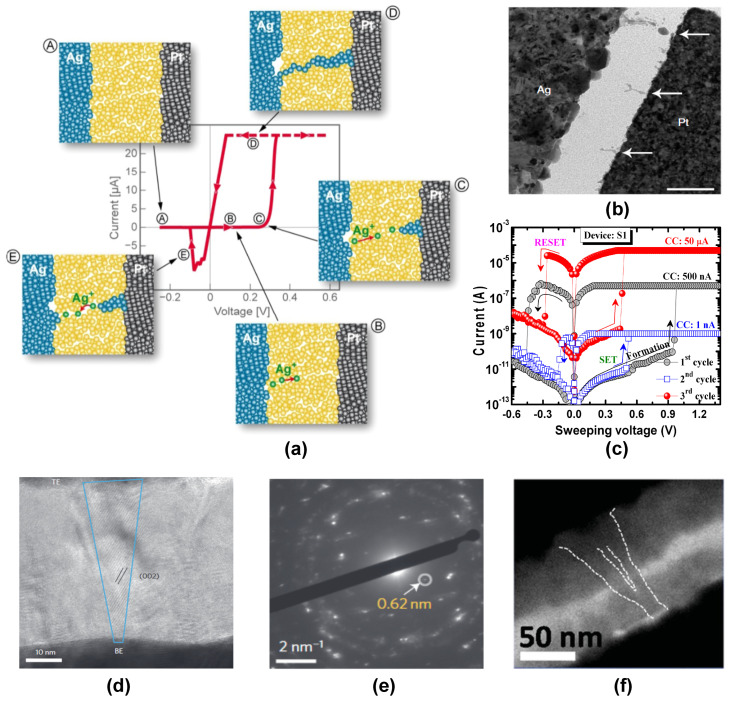
(**a**) Schematic showing the various steps involved in the SET and RESET processes of an ECM cell. Reprinted with permission from [29]. Copyright © 2024 IOP Publishing. (**b**) TEM image showing fully and partially formed Ag-conducting filaments inside a Ag/SiO_2_/Pt ECM cell. Reprinted with permission from [27]. Copyright © 2024 Springer Nature. (**c**) Typical bipolar switching characteristics observed in an ECM device. Here, the device configuration is Cu/GeO_*x*_/W. Reprinted under a Creative Commons License from [30]. (**d**) High-resolution TEM image of an oxygen rich conductive filament formed due to the formation of a Ti_4_O_7_ phase in a Pt/TiO_2_/Pt VCM device. (**e**) TEM diffraction pattern confirms the Ti_4_O_7_ phase formation. Reprinted with permission from [31]. Copyright © 2024 Springer Nature. (**f**) TEM image showing nanofilament formation by the oxygen vacancies in a Au/Ta_2_O_5_/Au VCM cell. Reprinted with permission from [32]. Copyright © 2024 John Wiley and Sons.

Ion-migration-based systems are the most prevalent switching mechanism observed in resistive switching devices. Ion migration can be based on both cation and anion migration to their respective opposite electrodes under the influence of an external field. Cation migration promoted switching is based on the phenomenon of electrochemical metallization (ECM), where the positive bias voltage applied to an electrochemically active metal electrode like Ag [26,27] or Cu [28] causes the movement of metal ions towards the counter electrode, which then leads to a redox reaction (see Figure 3a).

As the reduced metal atoms nucleate at the inert electrode, a metallic filament is slowly formed within the intermediate dielectric layer, which connects both the top and bottom electrodes. Once the filamentary connection is established, the device is set into a low-resistance state (LRS). On applying a reverse voltage, the rupture of the conductive filament leads to the device regaining its original high-resistance state (HRS). Factors like cation mobility, crystal structure of the dielectric [33], and the rate of redox reaction play an important role in determining the nature of filament formation and density of filaments, respectively. ECM-based devices have the advantages of low operation voltages, a large ON/OFF ratio, and scope for large-scale integration, but they also suffer from low-retention time [34]. Yang et al. [27] used an SiO_2_/Ag device to reveal nanoscale metallic filaments inside the SiO_2_ dielectric using a transmission electron microscopy (TEM) system (see Figure 3b). They could verify the theoretical ECM predictions of metallic dendrites formed from the inert electrode to the active electrode. In order to further confirm the role of cation mobility in the filament formation, the group also fabricated a similar device based on amorphous silicon (*a*-Si) instead of SiO_2_ as the switching layer. Due to the lower cation mobility, it was found that the *a*-Si films required a higher electric field for the forming process. T. Tsuruoka et al. [35] studied the cation migration in a resistive switching device having the composition Cu/Ta_2_O_5_/Pt. On application of a positive bias voltage, Cu^2+^ ions migrated towards the Pt electrode. Due to the anodic reduction reaction at the counter electrode, the Cu^2+^ ions nucleate inhomogeneously at the Pt electrode and form a conductive bridge towards the Cu electrode, achieving the LRS or SET state. When a negative bias is applied to the Cu electrode, due to the Joule heating at the filament, the Cu filament is disconnected, and hence the HRS or RESET state is achieved. S.Z. Rahaman et al. [30] compared the performance of Cu and Al electrodes on the switching performance of GeO_*x*_/W crosspoint RRAM devices and confirmed that the Cu electrode can produce low current switching varying from 1 nA to 50 μA and at lower operation voltages as compared to the Al-based system (see Figure 3c). Recently, J.H. Yoon et al. [36] used a Ruthenium-based resistive switching device to show the mechanism of Ru cation migration during the filament formation process. In situ TEM and ex situ EDS mapping were used to image the Ru-conductive filament formed due to the diffusion of Ru ions from the bottom electrode to the oxide medium. The device exhibited excellent switching characteristics, including low switching current (<1 μA), fast switching speed, and long retention. By using a temperature-dependent current-voltage (I–V) measurement analysis, hopping, and tunneling conduction were revealed in HRS and LRS states. Similarly, M. Luben et al. [37] compared various active electrode materials like Au, Ag, Al, Cu, Ni, Fe, Ti, Ta, V, and Zr for their electrode behavior in resistive switching applications. It was found that the electrochemical behavior of the active metal plays the most important role in determining the switching efficiency, filament stability, and longevity of the device. They quantified the best-performing electrode material using the Gibbs free energy of formation which was found to have an optimal value slightly above 0 kJ mol^−1^. Other factors like alloyed metal electrodes [38] and the effect of electromigration [39] have also been reported in recent literature.

Anion migration [40] is another kind of ion migration mechanism in resistive switching devices, where typically filaments are formed due to the increased concentration of oxygen vacancies within the oxide switching medium. This type of switching mechanism is generally referred to as the valence change mechanism (VCM). In general, these devices do not require significant electrochemical differences between the two electrodes, and hence, mostly inert electrodes of the same nature are generally preferred as the top and bottom electrodes. An essential requirement for the initiation of anion migration is the formation process, which switches the device to the LRS state. During the forming process, the high electric field applied to the device pushes the oxygen ions from the oxide layer towards the anode, creating localized oxygen vacancies inside the switching layer. These oxygen vacancies create a conductive filament joining the anode and the cathode, thereby resulting in the resistive switching process (see Figure 3d,e). On applying a negative voltage, the oxygen anions migrate back to the switching medium, thereby annihilating with the oxygen vacancies. This leads to a rupture of the filament, leading to the attainment of the HRS state. J.Y. Chen et al. [32] studied the oxygen vacancy rich filament growth inside a Au/Ta_2_O_5_/Au VCM cell and was successful in visualizing the filament evolution at various switching stages (see Figure 3f). In VCM devices, filament formation requires a high initial voltage, which results in the electroforming process. The high voltage required for the electroforming process has been observed to be disadvantageous for practical computing applications. Hence, a bilayer architecture [41] has been suggested, which has an oxygen-deficient layer and an oxygen-rich layer. Recently, R. Zhang et al. [42] used a flexible RRAM device based on a bilayer architecture of TiO_2_/HfO_2_ to study the oxygen vacancy distribution inside the system. By using XPS measurements, they were able to reveal the asymmetric hour-glass-shaped oxygen vacancy distribution at the interface between TiO_2_ and HfO_2_, which plays a major role in the filament formation and rupture processes. The coexistence of both metallic and anion-migration-based filaments was reported by Sun et al. [43] on a perovskite-based RRAM device having the structure Ag/CH_3_NH_3_PbI_3_/Pt. They observed that as the perovskite thickness is close to 300 nm, the switching is dominated by the iodide-vacancy-based VCM mechanism because of the incomplete formation of Ag conductive filament between the top and bottom electrodes. As the thickness of CH_3_NH_3_PbI_3_ film was reduced below 90 nm, the switching mechanism was dominated by ECM due to the improved conductivity of the fully formed Ag filaments. An additional variation in the VCM/ECM mechanism is observed in some devices, where the rupture of the filament occurs due to the Joule heating of the filament. These are called thermochemical mechanism (TCM) [44] based RRAM devices. Recently, X. Zhang et al. [45] studied the effect of Joule heating on the switching mechanism in a Pt/Al/AlO_*x*_/ITO-based device showing both unipolar and bipolar characteristics. It was observed that for unipolar switching, the high current flow through the filament increases the temperature of the filament beyond its critical temperature, thereby rupturing it. This effect switches the system to the RESET state. For the bipolar switching, the high reverse current causes the oxygen anions to migrate back to the AlO_*x*_ layer in addition to the Joule heating effect, thereby causing the filaments to melt down and pushing the system towards the RESET state. Y. Wang et al. [46] reported an electroforming free VCM cell with the configuration of W/ZnO/LTO/TiN with a lanthanum titanium oxide (LTO) switching layer. The addition of an oxygen-deficient ZnO layer provided additional oxygen ion migration pathways as well as an oxygen reservoir, resulting in an increased ON/OFF ratio as well. Theoretical modeling attempts to understand the switching mechanism in VCM devices have been reported recently by M. Kaniselvan et al. [47]. They used a combination of stochastic kinetic Monte Carlo methods in combination with quantum transport models with inputs from density functional theory calculations to investigate the effect of interface interactions between the oxide layer and the metal atom. In conclusion, continued research on the deeper understanding of ion migration is vital for the development of RRAM devices that are both high-performing and durable, aligning with the evolving demands of next-generation computing technologies.

#### 3.2.2. Trap Assisted Switching

**Figure 4:**
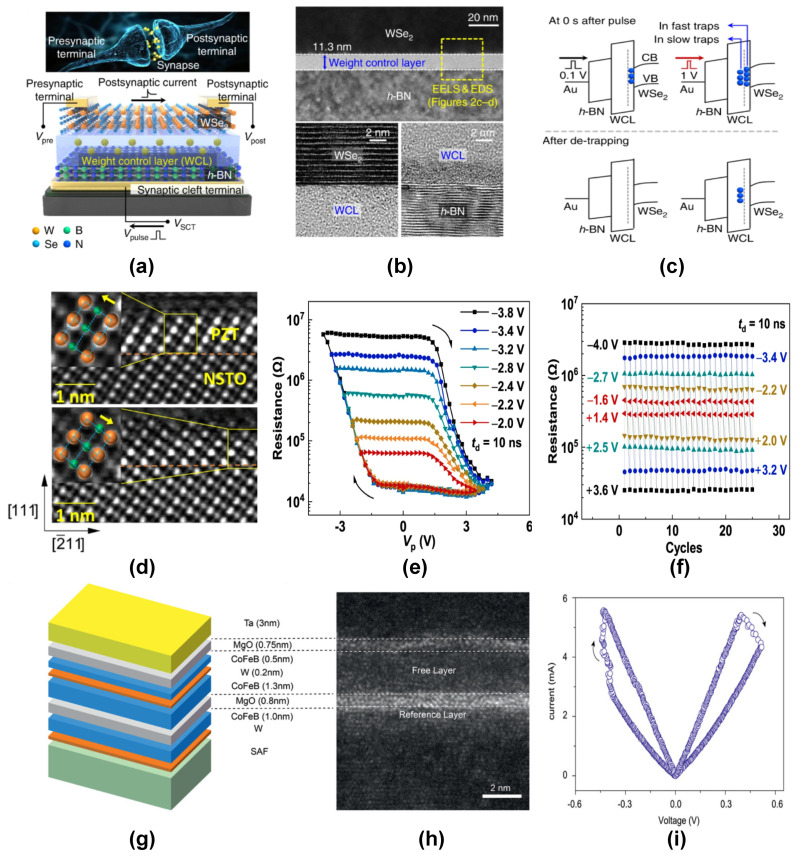
(**a**) Schematic of a WSe_2_/WCL/h-BN memristor device, along with the biological synapse comparison. (**b**) Cross-sectional TEM image of the WSe_2_/WCL/h-BN device structure. Lower insets show the magnified images for the WSe_2_/WCL and WCL/h-BN interfaces. (**c**) Energy band diagram for carriers in trap states demonstrated in a WSe_2_/WCL/h-BN device. Reprinted with permission under a Creative Commons CCBY License from [50]. (**d**) Opposing atomic displacements of the ferroelectric PZT/NSTO device recorded using the cross-sectional HAADF-STEM images. Inset shows the schematic of the Pb (orange spheres) and Zr/Ti ions (green spheres) with opposing polarisation directions. (**e**) Resistive switching observed in the FTJ device Ag/PZT/NSTO with respect to the applied pulse voltage at various maximum negative voltages. (**f**) Endurance characteristics of the FTJ device for different pulse voltage, and a pulse duration of 10 ns. Reprinted with permission under a Creative Commons CCBY License from [58]. (**g**) Device structure of the MTJ device and its (**h**) cross-sectional TEM image showing the various device layers. (**i**) Analog switching behavior of the MTJ device for an applied pulse width of 200 ns. Reprinted with permission under a Creative Commons CCBY License from [62].

The charge trapping-detrapping mechanism is an electronic switching mechanism that is based on the trapping and deptrapping of electrons in the trap states inside the dielectric film. Since this is a purely electronic process, there are no microstructural changes [48] inside the dielectric film during the resistive switching process. This is highly advantageous compared to the ion migration mechanism, where the changes in crystalline morphology induce unwanted performance variations during device-to-device characterization. X.F. Cheng et al. [49] reported the trapping/detrapping mechanism in a 1D d-*π* conjugated coordination polymer chain-based resistive switching device exhibiting a multilevel switching behavior. It was found that the traps originating from impurities or structural defects within the polymer material gave rise to a trap-assisted conduction region in the logarithmic I–V plots, where the current I was found to be proportional to the voltage squared. S. Seo et al. [50] used a h-BN/WSe_2_ vanderwaals heterostructure device to modulate the synaptic weight of the neuromorphic device using an additional weight control layer (WCL) formed over the h-BN layer by O_2_ plasma treatment (see Figure 4a–c). The trap states in the WCL layer were found to determine the conductance of the synaptic weights. Charge trapping-detrapping-based conduction switching was demonstrated in graphene quantum dots (GQD) by H.Y. Choi et al. [51] using a PEDOT:PSS:GQD/Al device. Using a UV-photoelectron spectroscopy study and band structure analysis, the group could identify the space charge-limited conduction (SCLC) mechanism due to the trap states in the GQDs which were responsible for the switching behavior. Recently, S. Ganeshan et al. [52] reported the charge trapping/detrapping mechanism in a water-soluble MoS_2_ quantum dot (QD)/PVA-based device with copper electrodes. By analyzing the log I–log *V* characteristics and the band diagram of the device, they could clearly identify the charge trapping behavior of MoS_2_ QDs, which were responsible for the switching characteristics. In summary, the charge trapping-detrapping mechanism, by avoiding microstructural changes unlike ion migration, provides a stable and efficient switching mechanism for resistive switching memories, as evidenced by recent advancements in materials and device engineering.

#### 3.2.3. Other Prominent Mechanisms

Apart from the above mechanisms, several other mechanisms can indirectly result in resistive switching. These include ferroelectric polarization switching, spin-torque-based switching, ion intercalation, etc. Table 2 summarizes the comparison of performance metrics of various resistive switching mechanisms. Resistive switching devices based on ferroelectric polarization switching have received extensive attention due to their long retention times [53,54] and forming free switching behaviours [55]. Ferroelectric tunnel junction (FTJ)-based memristors [56] consist of a thin ferroelectric barrier layer inserted in between two metal electrodes. The device exhibits resistance states depending upon the direction of electric polarization and domain configurations. C. Wang et al. [57] reported the ferroelectric resistive switching in an epitaxially grown BiFeO_3_(BFO) thin film sandwiched between SrRuO_3_ and Pt electrodes. The switchable diode effect exhibited by the device was found to be due to the Schottky-like barriers, which were modulated by the ferroelectric polarization direction and oxygen vacancies. Recently, Z. Luo et al. [58] demonstrated a FTJ-based RRAM device based on the Ag/PZT/Nb_0.7_:SrTiO_3_ heterostructure (see Figure 4d–f). By utilizing an ultrathin (∼1.2 nm) layer of (111)-oriented PZT ferroelectric ferroelectric barrier layer, the device exhibited 150 reproducible conductance states, with a high switching endurance of 10^9^ cycles.

The manipulation of the magnetization of a material using spin transfer torques [59], current injection [60], etc. has been found to be useful for resistive switching applications. Although the underlying mechanism behind such resistance switching mechanisms might be magnetic in origin, the effect of such magnetic property manifestation can result in a change in the resistance state of the device. Hence, magnetic property-based switching phenomena can also be considered as an underlying mechanism responsible for resistive switching. P. Krzysteczko et al. [61] demonstrated various synaptic plasticities like LTP, LTD, and STDP using a magnetic tunnel junction (MTJ)-based resistive switching device. By using a MgO tunnel barrier for the device, they found that the MTJ resistance changed with continuous application of voltage pulses.

**Table 2:** Comparison of performance metrics of various switching mechanisms.

Switching Mechanism	Device Structure	$R_{\mathrm{OFF}}$/ $R_{\mathrm{ON}}$	Endurance (Cycles)	Retention (s)	Operating Voltage (V)
Electrochemical Metallization (ECM)	Ag/*a*-ZnO/Pt [63]	>10^7^	>10^2^	>10^6^	<1
Valence Change Mechanism (VCM)	Ti/HfO_2_/TiN [64]	$10^{5}$	$10^{10}$	$10^{4}$	3
Trap Assisted Switching	Nb/NbO_x_/Al_2_O_3_/HfO_2_/Au [65]	$10^{2}$	>10^2^	>10^5^	3.7
Ferroelectric Polarization	Ag/PZT/Nb:SrTiO_3_ [58]	>10^2^	$10^{9}$	$10^{4}$	1.35–2
Magnetization Reversal	W/CoFeB/MgO/CoFeB/IrMn [66]	∼10^2^	$10^{12}$	>10^6^	−
Metal Insulator Transition	Pt/Al/PCMO/Pt [67]	>10^2^	$10^{6}$	>10^6^	3
Ion Intercalation	Ni/LiCoO_2_/*a*-Si/Ti [68]	∼10	−	∼10^4^	8

X. Zhang et al. [62] reported a nanoscale spin-torque memristor with the configuration Ta/MgO/CoFeB/W/CoFeB/MgO/CoFeB/W (see Figure 4g,h). Here, the CoFeB/W/CoFeB composite layer is a perpendicular anisotropy MTJ with the free W layer. The analog switching characteristics in this MTJ device (see Figure 4i) originates from the strong magnetic domain wall pinning phenomenon in the W layer. Similarly, Y. Wei et al. [69] integrated a Hf_0.5_Zr_0.5_O_2_ (HZO) tunnel barrier between two ferromagnetic electrodes to form a multiferroic tunnel junction (MFTJs) device, which was able to demonstrate four non-volatile resistance states. The stable resistance states were obtained by external electric and magnetic fields by using a combination of tunnel electroresistance (TER) and tunnel magnetoresistance (TMR). Recently, magnetic memristors based on the concept of spin torque oscillators have been widely reported for their high efficiency in spoken digit recognition [70]. Similarly, analog memristors based on magnetic domain walls [71] and skyrmion dynamics [72] have also been found to be efficient neuromorphic devices. Metal-insulator transition has been extensively studied in many novel materials, like VOx [73], NbOx [74], etc. Mott insulators [75] are of special interest among materials exhibiting MIT transition due to their sharp ON/OFF transition, which can be electrically modulated. Recently, X. Zhang et al. [76] demonstrated an artificial afferent spiking neuron device using a NbOx mott memristor. They could control the spiking frequency of the afferent neuron device in proportion to the input stimuli intensity. This spiking behavior was reversed to lower frequencies when encountering abnormally high input stimuli. These neuronal responses are similar to the nervous responses in biological systems, paving the way for developing future neurorobotic devices.

Ion intercalation is another type of mechanism responsible for the switching behavior in electrolyte-based synaptic devices. The mobile ions in the liquid electrolyte alter the band structure of the dielectric switching layer, which changes the conductance of the system. E. J. Fuller et al. [77] employed a three-terminal electrochemical resistive switching device based on LiCoO_2_ switching layer. The conductance of the LiCoO_2_ layer was found to depend on the amount of Li intercalation. C. S. Yang et al. [78] used an Ag/MoO_x_/FTO based resistive switching device to study the interfacial electrochemical reaction occurring in the MoO_x_ films adsorbed with water. They identified that the application of an electric field generates protons due to the decomposition of the adsorbed water molecules. These protons are intercalated into the MoO_x_ lattice, which gives rise to different synaptic behaviors under various applied bias voltages, pulse numbers, pulse frequency, etc. Recently, X. Yao et al. [79] demonstrated proton intercalation in a WO_3_-based three-terminal resistive switching device exhibiting long retention characteristics and good reproducibility. By utilizing XPS measurements and density functional theory (DFT) analysis, they could assess the role of mobility and carrier density in modulating the conductance of protonated WO_3_. A gate-controlled iontronic memtransistor device based on a bilayer thin film of poly(ethylene)oxide(PEO), and rubidium silver iodide (RbAg_4_I_5_) was recently reported by A. Mukherjee et al. [80], where the Ag^+^ ionic movement inside the RbAg_4_I_5_ layer was found responsible for the resistive switching mechanism. Interestingly, the device exhibited rich physics, like colossal hysteresis and negative differential transconductance, revealing interesting research possibilities in these devices. In summary, indirect mechanisms, like ferroelectric polarization switching, MTJ-based switching, domain wall dynamics, ion intercalation, etc., need to be studied further and can also be responsible for resistive switching in neuromorphic devices.

## 4. Resistive Switching Materials & Applications

The material selection for a resistive switching device plays an important role in determining the various performance parameters like switching voltage, ON state resistance, ON/OFF ratio, retention, and endurance characteristics of the device. Traditionally, all-inorganic systems were identified as the preferred choice of the switching layer in resistive switching devices due to their reproducible switching behavior, and ease of fabrication. Inorganic oxides, including binary oxides, complex oxides, and oxide heterojunctions, have been among the most widely used materials for RRAM applications. Apart from oxide materials, various other inorganic compounds, like chalcogenides and nitrides, have also been studied. Later, organic materials, including small molecules, polymers, and biomaterials, have been used for their flexibility and biodegradability. Currently, lower-dimensional structures, including nanoparticles, nanowires, 2D materials, quantum dots, and van der Waals (vdW) heterostuctures, are finding huge interest due to their atomic-level sizes and tunable electronic properties, which are both advantageous for neuromorphic emulation. Here, we discuss the various material choices for RRAM devices and their neuromorphic capabilities.

### 4.1. Inorganic Materials

#### 4.1.1. Oxide Materials

**Figure 5:**
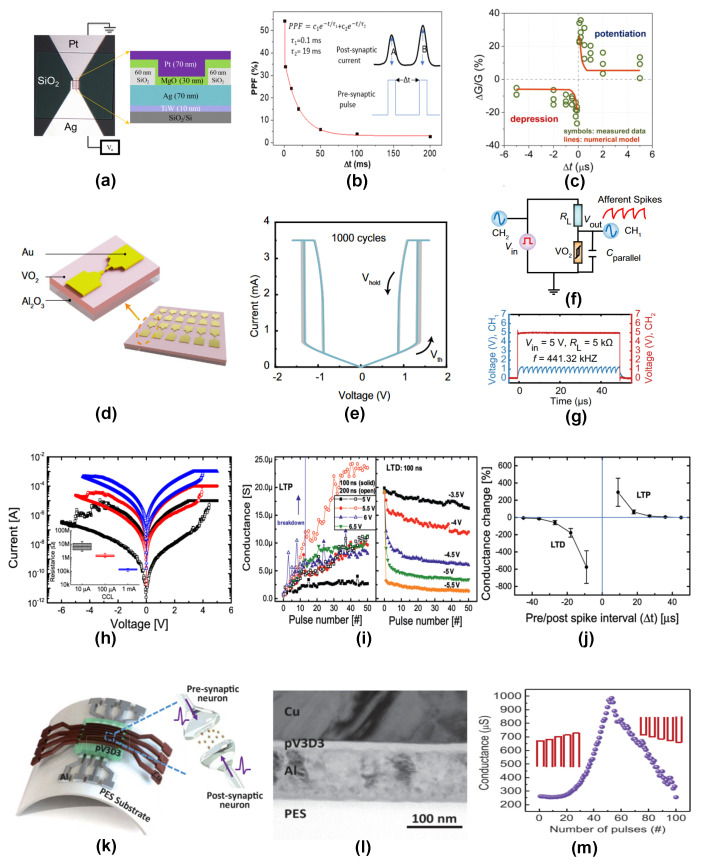
(**a**) Optical image & device schematic of Ag/MgO/Pt RRAM device. (**b**) Paired pulse facilitation (PPF) achieved using the Ag/MgO/Pt-based RRAM device. Reprinted with permission from [84]. Copyright © 2024 AIP Publishing. (**c**) Experimental and simulated STDP curves obtained in a second-order memristor device. Reprinted with permission from [86]. Copyright © 2024 American Chemical Society. (**d**) Schematic of the epitaxial VO_2_-based RRAM device stack. (**e**) Threshold switching characteristics of the RRAM device. (**f**) Electrical circuit & (**g**) LIF response observed using a threshold switching RRAM device. Reprinted with permission under a Creative Commons CCBY License from [87]. (**h**) Compliance current controlled analog switching characteristics of a Ni/SiN_*x*_/AlO_*y*_/TiN RRAM device. (**i**) Conductance change during LTP & LTD while applying identical pulse voltages. (**j**) STDP response of the device. Reprinted with permission from [89]. Copyright © 2024 American Chemical Society. (**k**) Schematic of a pV3D3-based flexible memristor array. (**l**) Cross-sectional TEM image showing the device stack of CU/pV3D3/Al configuration. (**m**) Potentiation/Depression characteristics of the flexible RRAM device. Reprinted with permission from [13]. Copyright © 2024 American Chemical Society.

Oxide materials have been the default choice of resistive switching material for neuromorphic applications. Among the various advantages of these oxides, their CMOS technology compatibility and inexpensive fabrication techniques have made these materials ideal choices. T. W. Hickmott et al. [81] reported the first resistive switching characteristics using a binary oxide of Al_2_O_3_ sandwiched between two aluminum electrodes. Later, several different groups explored the synaptic capabilities of binary oxide-based memristor devices. M. Cavallini et al. [82] reported a CMOS-compatible Si/SiO_2_/Al RRAM device with a high ON/OFF ratio of $10^{5}$. The SiO_2_ layer in this device has been fabricated in situ by using a local oxidation lithography process. The device offered several advantages, like a fully regenerable junction, spatially controllable dielectric layer patterning, and a novel in situ fabrication procedure that prevented the chances of cross-talk through the dielectric thin film. S. Yu et al. [83] used a multilayer memristor device based on HfO_*x*_/AlO_*x*_ to demonstrate the synapse capabilities. The device recorded sub-picojoule energy consumption with high endurance and retention characteristics. By using a time domain multiplexing (TDM) approach to convert the difference in spike timing to the pulse amplitude difference, the STDP synapse behavior was achieved in this device. Y. Zhang et al. [84] designed a Ag/MgO/Pt resistive switching device (see Figure 5a,b) which exhibits a volatile switching behavior. By varying the input pulse parameters like pulse amplitude, pulse interval, etc., they were successful in demonstrating various synaptic functionalities like paired pulse facilitation (PPF), LTP, and a reversible transition from STP to LTP. It was found that the formation and sudden breakage of the silver filaments due to varying applied pulse stimuli was the underlying mechanism responsible for the various synaptic responses. Recently, M. Rao et al. [85] reported a bilayer device with HfO_2_/Al_2_O_3_ as the switching layer and Ti/Ta, Pt as the top and bottom electrodes, respectively. The device reported a record number of 2048 reproducible conductance states with remarkable linearity and CMOS integration capability. At the time of publication of this report, this device has reported the highest number of conductance levels from an RRAM device. S. Kim et al. [86] used a second-order memristor (see Figure 5c) based on a resistive switching layer Ta_2_O_5−*x*_ along with a conductive TaO_*y*_ film that acts as the oxygen vacancy reservoir. The device functions based on the modulation of the internal state variables to encode information on spike timing and synapse activities. The study could reveal that the second-order state variable temperature played an identical role to the Ca^2+^ dynamics in biological synapses. By carefully designing a spike signal composed of a programming signal and a heat pulse, they were able to demonstrate the STDP behavior in the system. R. Yuan et al. [87] reported a calibratable neuron device (see Figure 5d–g) based on a planar structure of Au/Ti/VO_2_/Ti/Au fabricated on Al_2_O_3_ substrate. They could reproduce the leaky integrate-and-fire (LIF) response of a sensory neuron using the threshold switching characteristics of the VO_2_ device. Metaplasticity was demonstrated on a WO_3_-based memristor by Z. H. Tan et al. [88]. They could clearly establish the effect of the stimulus history and time interval on the STDP behavior of the memristor device.

Recently, H.G. Hwang et al. [90] demonstrated metaplasticity effects using a Ta_2_O_5_ memristor device. Using a preliminary spike in addition to the pre- and post-synaptic spikes, the device was successful in emulating the metaplasticity of STDP behavior. In addition, the same device was also able to exhibit other plasticities like the STP-to-LTP transition and SRDP. Similarly, heterosynaptic plasticity was demonstrated using a four-terminal TiO_2−*x*_ [91] memristor device. By employing oppositely arranged pairs of electrodes for read/write and gate operations, the device exhibited the gate tuning of potentiation and depression behaviors of a synapse junction. In addition to binary oxides, complex oxides like InGaZnO [92], LaAlO_3_ [93], BiFeO_3_ [94], SrTiO_3_ [95], KNbO_3_ [96], HfZrO_*x*_ [97], SiO_*x*_N_*y*_ [98] etc. have also been extensively studied for their neuromorphic applications.

#### 4.1.2. Other Inorganic Materials

Apart from oxide materials, various other inorganic materials, including nitrides [89], sulphides [99], phosphides [100], tellurides [101], and chalcogenides [102], have also been studied for their synaptic functionalities. S. Kim et al. [89] demonstrated various synaptic responses, including LTP, LTD, and STDP, by adjusting the pulse amplitudes and timing sequence of the pre- and post-spikes applied to a silicon nitride (SiN_*x*_)-based memristor device (see Figure 5h–j). Unlike some of the oxide materials, SiN_*x*_ has the added advantage that it is fully compatible with the CMOS technology, and hence it is easily scalable for industrial production. Recently, Y. Guo et al. [103] fabricated an aluminum nitride (AlN)-based memristor device using a reactive magnetron sputtering technique. Pt and TiN were used as the bottom and top electrodes, respectively. LTP and LTD plasticities were successfully demonstrated by varying the compliance current levels, stop voltages, and pulse modes applied to the memristor device. Similarly, H. Cho et al. [104] utilized an AlN memristor to emulate LTP-to-STP transition and PPF by modulating the pulse interval time and pulse number. L. Hu et al. [99] reported memristors based on lightly oxidised ZnS films, which were found to have ultra-low SET voltage and stable resistive switching characteristics. In addition to the LTP and STP responses, the device could successfully replicate dynamic neural functions like memorizing and forgetting. M. Chen et al. [100] reported a cuprous phosphide (Cu_3_P) based RRAM device with a high ON/OFF ratio of $2.1\times 10^{4}$ using a nickel top electrode. The switching mechanism was reported to be due to the redox reactions involving Cu^2+^ and P^3−^ ions at the electrode interfaces. Y. Sun et al. [105] used a memristor system based on Ag/GeSe/TiN for mimicking electronic synapses. Spiking pulses of a few hundred millivolts were used to reproduce various synapse responses like STP, LTP, PPF, and STDP. Y. Li et al. reported chalcogenide memristors based on Ge_2_Sb_2_Te_5_ [102] and AgInSbTe [106], which were capable of achieving four different types of STDP behaviour depending on the spiking protocol of input pulses. Another important class of materials relevant to memristor applications are inorganic halides like iodides [107] and bromides [108], which have also been extensively studied for neuromorphic emulation.

### 4.2. Organic Materials

#### 4.2.1. Polymer Materials

The ecological problems arising from the excessive use of inorganic switching materials and newer requirements for biodegradable devices have pushed the research towards developing alternate organic-based devices for several conventional devices like transistors [109], batteries [110], solar cells [111], LEDs [112], etc. A number of RRAM devices have also been reported based on unique organic-material-based device designs [113]. Polymers are a class of organic materials which have the advantages of low toxicity, high flexibility, low power consumption, and excellent biocompatibility. S. Li et al. [114] reported the first two terminal polymer memristor device capable of emulating synaptic responses. They used an Ag/poly(3,4-ethylenedioxythiophene):poly(styrenesulphonate)(PEDOT:PSS)/Ta stacked device. Various synaptic plasticities like STP, LTP, STP to LTP, STDP, and SRDP were successfully demonstrated using this simple and low-cost device configuration. The device exhibited a rectification effect, which was found useful in emulating the direction-dependent information flow across biological synapses. By using a combination of cross-sectional TEM and energy-dispersive X-ray spectroscopy, the movement of the Ag interface due to the redox reaction induced by the applied electric field and the elastic effect of the PEDOT:PSS medium were identified as the physical mechanisms responsible for the synapse response. Similarly, Y.V. de Burgt et al. [115] reported an electrochemical neuromorphic organic device composed of a PEDOT:PSS presynaptic electrode and PEDOT:PSS/poly(ethylenimine) (PEI) composite post-synapse electrode. The device could switch its conductance at very low voltages (∼10 pJ) comparable to their commercial inorganic counterparts. In addition to their learning capabilities and image recognition features, these organic memristors were capable of producing LTP and LTD plasticities with over 500 distinct conductance states over their operating voltage range. Recently, B. C. Jang et al. [13] fabricated a flexible memristor based on poly(1,3,5-trivinyl-1,3,5-trimethyl cyclotrisiloxane) (pV3D3) sandwiched between Cu and Al electrodes (see Figure 5k–m). The switching behavior of the memristor was found to change from binary switching to analog synaptic switching based on the dimensions of the conducting filament. The device was found to be capable of imitating several synapse behaviors like potentiation/depression, PPF, and STDP. By examining the ex situ TEM images, it was revealed that the analog switching and quantized conductance observed in the device was a result of the atomically thin part of the Cu filament formed inside the memristor device. T.F. Yu et al. [116] developed a field effect transistor based memristor device with a *p*-type donor-acceptor conjugated polymer, poly-(thienothiophene-*co*-1,4-diketopyrrolo[3,4-c]pyrrole)(PDBT-*co*-TT) doped with an ionic additive tetrabutylammonium perchlorate (TBAP) as the active material. TBAP was used as the dopant mainly due to the high electron affinity of its constituent anions, which are expected to enhance the dopant-polymer interaction. The TBAP-doped devices exhibited a high memory window and an ON/OFF ratio of over $10^{3}$. The device was successful in emulating synaptic behaviors like EPSC, IPSC, and SRDP. A particularly notable feature of these devices is the high PPF index (204%) achieved using pulses of width 10 ms and pulse interval 10 ms. Lately, several other polymers like poly(11-(9H-carbazol-9-yl)undecyl methacrylate) (PUMA) [117], poly(3-(4^′^,4^‴^-dimethyl-[2^′^,2^″^:5^″^,2^‴^-terthiophene]-3^″^-yl)acrylic acid) (PMTAA) [118], poly-para-xylylene(parlyene) [119], poly(3-hexylthiophene) (P3HT) [7] etc. have also been studied for synaptic emulation.

#### 4.2.2. Biomaterials

Biocompatibility and flexibility are increasingly being looked upon as favorable traits for bio-integrated neuromorphic devices like medical implants and wearable electronics. Conventional RRAM materials based on inorganic oxides are not suited for such applications due to their toxicity. Biomaterials based on naturally available materials such as proteins [120], carbohydrates [121], DNA [122], RNA [123], virus [124], etc. are highly suited for bio-integrated applications and have been extensively studied as resistive switching materials. Recently, G. Wu et al. [125] used chitosan-based biopolysaccharide proton conductors as gate dielectrics for developing synaptic transistors on top of paper-based substrates. Chitosan is a linear polysaccharide whose proton conductivity can be greatly improved by acid doping. In this study, the high proton conductivity of chitosan achieved using acid doping was modulated with pulse voltages to emulate various synaptic responses. The fabricated devices could imitate EPSC, PPF, dynamic filtering, etc. Similarly, Yu et al. [126] fabricated a chitosan-gated oxide neuromorphic transistor that reproduces four types of STDP learning behaviors: Hebbian STDP, anti-Hebbian STDP, symmetrical STDP, and visual STDP. The device works on the basis of protonic doping and dedoping processes at the interface between the ITO gate and the Chitosan layer. Y. Park et al. [127] fabricated an artificial synapse device based on a natural polymer called Lignin, which is a common organic component found in natural wood. Lignin was spin coated on an ITO-coated flexible polyethylene terephthalate (PET) substrate along with an inert Au top electrode for the device fabrication. By applying predesigned pulse sequences of varying amplitudes and time scales, the memristor device could successfully emulate synaptic functions like EPSC, potentiation/depression, SRDP, and STP-to-LTP transition. It was concluded that the device operation in a lignin-based memristor was based on the behavior of carbon atoms in the lignin matrix. By varying the thermal energy applied using different pulse signals, the conductance of the lignin layer can be modulated due to the formation of amorphous carbon matrix- or graphitic-like structures in the active layer. Similarly, G. Wu et al. [128] used a naturally occurring protein in the form of chicken albumen to fabricate memristive devices with high proton conductivity. The chicken albumen was used as the electrolyte dielectric in indium-zinc oxide synaptic transistors. Various synaptic functionalities, including PPF, dynamic filtering, and STP-to-LTP transition, were also successfully mimicked using the fabricated devices. The synaptic behavior of the device is attributed to the modulation of the proton conductivity of the albumen film by the applied pulse voltages.

### 4.3. Lower Dimensional Materials

Lower-dimensional materials, including nanoparticles [98], quantum dots (QD’s) [129], nanowires [130], nanorods [131], carbon nanotubes (CNT) [132], and 2D materials [133] have been extensively studied for both resistive switching and neuromorphic applications. Z. Wang et al. [98] fabricated a new class of memristors for synaptic emulation based on the diffusion dynamics of Ag nanoparticles embedded inside the switching layer (SiO_*x*_N_*y*_). Using in situ HRTEM imaging, the microscopic mechanism responsible for the threshold switching mechanism was identified as the interfacial energy-driven diffusion mechanism of Ag nanoparticles inside the dielectric host lattice. The Ag nanoparticle dynamics inside the device were functionally similar to the Ca^2+^ dynamics inside the synapse and could faithfully emulate various synaptic responses like PPF, PPD, STDP, and SRDP. Recently, B. Salonikidou et al. [134] developed a fully printed memristor device based on a TiO_2_ nanoparticle ink formulation. These devices were fabricated using an inkjet printing technique and were found to be highly uniform and crack-free. The low electroforming voltage observed in the device might be attributed to the ease of conductive bridge formation within the TiO_2_ nanoparticle ink matrix. In addition, the device could successfully emulate the LTP and STP characteristics of a synapse system by controlling the trigger pulse rate and duration between pulse-interpulse at the input. T. Ishibe et al. [135] reported a Fe_3_O_4_/GeOx/Ge nanocrystal (NC)-based RRAM device exhibiting high switching probability (∼90%) and a high ON/FF ratio of ∼58. The device was composed of high-density and ultra-small Fe_3_O_4_ NCs grown on Ge nuclei deposited over a Si substrate. An interesting polymer-metal nanoparticle hybrid memristor was fabricated by S. R. Zhang [136] and team. They used a solution processed hybrid memristor with polyvinylpyrrolidone(PVPy)-Au nanoparticle (NP) composite as the active layer and Al and ITO as the top and bottom electrodes, respectively. The device exhibited good resistive switching properties and excellent artificial synapse responses. Various synaptic plasticities like SRDP, PPF, post-tetanic potentiation (PTP), STP-to-LTP transition, and learning- forgetting-relearning processes were achieved using this device. The trapping and de-trapping of charge carriers in the polymer-nanoparticle switching layer due to opposite-polarity input pulses was identified as the mechanism favoring the synapse response in these devices.

Recently, core-shell nanoparticles based on Au@Al_2_O_3_ [137] were also used to develop memristor devices with high stability and reliability. These devices were fabricated using atomic layer deposition (ALD) technique, where the Au@Al_2_O_3_ nanoparticles were grown in situ over the ITO substrate. Potentiation and depression responses of the device were attributed to the Fowler-Nordheim(FN)-tunnelling-mediated internal electric field developed in the Au@Al_2_O_3_ switching media. In addition to the dimensional reduction in nanoparticles, the confinement of electric field in Quantum-dot(QD)-based memristors was identified as a favorable attribute for synapse emulation. QD-based memristors for artificial synapse applications were first reported by X. Yan et al. [129], who used graphene oxide quantum dots (GOQD) as a composite with Zr_0.5_Hf_0.5_O_2_ (ZHO). The device used Ag as both the bottom and top electrodes, resulting in a Ag/ZHO:GOQD/Ag structure. The device possesses bidirectional control of resistance, which is a very important factor for the demonstration of synapse response. The device could demonstrate various synapse responses like STDP, PPF, and learning-experience behavior using very low pulse voltages (∼0.6 V) and pulse widths (∼30 ns).

**Figure 6:**
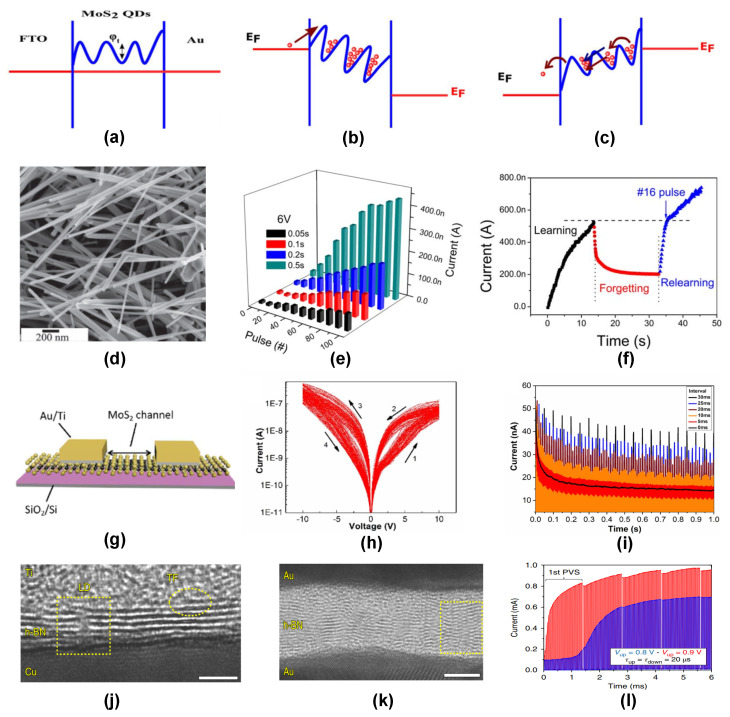
(**a**) Band diagram of a quantum-dotMoS_2_-based RRAM device showing the Schottky barrier at the Au/MoS_2_ interface and trap states in the MoS_2_ layer. (**b**) Electron trapping in the defect sites under a positive bias voltage. (**c**) Electron detrapping due to negative bias voltage. Reprinted with permission under a Creative Commons CCBY License from [139]. (**d**) SEM image of TiO_2_ nanowires in an Au/TiO_2_/Au RRAM device. (**e**) Potentiation observed in the TiO_2_ nanowire RRAM for different pulse amplitudes & widths. (**f**) Sequence of learning-forgetting-relearning process observed in the same device. Reprinted with permission from [140]. Copyright © 2024 IOP Publishing. (**g**) Schematic of the two-terminal MoS_2_-based device. (**h**) Analog switching characteristics exhibited by the same device for 100 I–V cycles. (**i**) Low pass filtering behavior achieved in the device using −10 V, 20 ms width pulses of different pulse intervals. Reprinted with permission from [133]. Copyright © 2024 IOP Publishing. Cross-sectional TEM image of vdW heterostructure h-BN devices with (**j**) Au/Ti/5–7 layer h-BN/Cu (**k**) Au/15–18-layer h-BN/Au configurations. (**l**) Potentiation achieved in a 5 μm×5 μm, Au/Ti/5–7-layer h-BN/Au device by applying two sequences of pulsed voltages of amplitude ($V_{up}$) −0.8 V (blue) and 0.9 V (red) with pulse period 20 μs. Reprinted with permission from [144]. Copyright © 2024 Springer Nature.

The action of electochemical metallization of Ag^+^ ions at lower electric fields and the combination of FN tunneling and direct tunneling at higher electric fields were found to dominate the memristive behavior of the system. The same group has also reported a similar study [138] based on lead sulfide (PbS) QD-based memristors capable of emulating similar synapse functionalities. Recently, MoS_2_ QDs were used as the active layer for memrstive synapse emulation by A. Thomas et al. [139] (see Figure 6a–c). They used a liquid-phase exfoliation method to synthesize MoS_2_ QDs in order to fabricate a memristor with the configuration of Au/MoS_2_ QD/FTO. The device was successful in demonstrating STP behaviors like PPF and PPD due to the trapping/detrapping of charge carriers in the QD defect sites as a result of the applied pulse polarities. As compared to nanoparticles and QDs, nanowires have the additional advantage that they are highly confined, and hence straight conduction paths are easily established, which would improve the carrier transport properties of the device.

Nanowire-based memristive devices for synaptic applications were first reported by Hong et al. [130] in a TiO_*x*_ nanowire-based device. They fabricated TiO_*x*_ nanowires using an electrospinning method and transferred a single nanowire to an SrTiO_3_ substrate with the same SrRuO_3_ (SRO) electrode acting as the anode and cathode in a planar architecture. Although the device requires a very high switching voltage of 70 V, the device could exhibit various synaptic plasticities like STDP, anti-STDP, and Hebbian learning rules. The redistribution of the oxygen vacancies in the TiO_*x*_ nanowire due to the polarity of the applied electric field was identified as the mechanism responsible for the synapse response. Similarly, B. Zhao et al. [140] fabricated a TiO_2_ nanowire-based memristor device (see Figure 6d–f), which could switch the conductance at considerably lower voltages. They used a technique called dielectrophoresis for the device fabrication. SRDP and learning-forgetting-relearning behavior were demonstrated in the nanowire-based device using programmed pulse voltages. The synaptic learning response originated due to the oxygen vacancy migration caused by the applied electric fields. As the pulses are removed, reverse diffusion of the oxygen vacancies and electron trapping leads to metastable oxygen lattices, which can be easily separated by applying lower voltages during the relearning process. K. Nagashima et al. [141] reported multistate bipolar resistive switching in a single core/shell nanowire of MgO/Co_3_O_4_. The device exhibited a remarkable endurance of $∼\;\;10^{8}$ mediated by a voltage controlled switching mechanism. A ZnO nanowire device [142] with an unltrathin TiO_*x*_ interfacial layer was also reported recently, which could emulate several short-term plasticity behaviors such as PPF, PPD, etc. The interfacial layer minimized the effects of surface defects in the ZnO nanowire and assisted in electron hopping at lower electric fields and barrier tunneling at higher electric fields. Recently, Y. Choi et al. [132] fabricated a carbon-nanotube(CNT)-based memristor device capable of simulating various synaptic plasticities. The device has a transistor structure, where a ferroelectric polymer is capacitively connected to a gate dielectric (SiO_2_) through a single-walled carbon nanotube (SWCNT). The electric field permeability of the SWCNT enabled the remote control of the ferroelectric polarization, which controlled the synaptic weights. By adjusting the synaptic weight updates, various synaptic responses like STP, LTP, LTD, etc. were observed. Similarly, CNTs were used in several recent studies [143] to emulate similar synaptic behaviors.

Currently, two-dimensional (2D) materials have gained a lot of interest in RRAM based neuromorphic devices, mainly due to their atomically thin dimensions, low power consumption, and the possibility of fabricating sub- 10 nm-channel-length devices. Further, 2D materials have tunable electronic properties like band structure, carrier mobility, etc., which depend on the number of layers of the material. Such adjustable electronic features are highly suited for the emulation of neurological responses. Graphene [145] has been the most widely studied 2D material, mainly due to its unique electronic and mechanical properties. H. Tian et al. [146] reported the first graphene-based synaptic device having a standard FET configuration and an additional bottom gate electrode. The device used twisted bilayer graphene channels with gold electrodes as the source and drain and Al SiO_2_ as the top and bottom gate electrodes, respectively. The migration of the pulse-generated oxygen anions in the oxidised Al electrode leads to changes in the current through the graphene layer, which helps in mimicking the various plasticity behaviors. In addition, the bottom gate acts as a control element to modulate the plasticity even further. The device could reproduce several plasticity responses, like LTP, LTD, and STDP, with very low excitation pulses. Recently, J Shen et al. [133] (see Figure 6g–i) demonstrated a multi-state LTP response using a single-crystal-monolayerMoS_2_-based memristor device. The device achieved a very low power consumption of 1.8 pJ after LTP, which is the lowest among other reported MoS_2_ devices. The mechanism responsible for the synapse behavior was identified as the sulfur vacancy migration under the applied voltage pulses, which caused variations in the Schottky barrier height at the MoS_2_/metal interface, leading to the synaptic weight modulation. 2D MoS_2_ was recently used by D. Devet al. [147] to fabricate a volatile threshold switching RRAM device with a high ON/OFF ratio of $10^{6}$. The threshold switching RRAM device was used in parallel to a capacitor to implement a leaky integrate-and-fire (LIF) neuron circuit. A similar LIF circuit based on threshold switching 2D graphene memristor device was also recently reported [148].

Van der Waals (vdW) heterostructures [149] are a relatively new class of low-dimensional materials where multiple 2D materials are stacked vertically on top of another and held together by weak van der Waals interaction. Due to their reduced dimensions and dangling bond-free surfaces, they are ideal candidates for large-scale integrated artificial synapses for hardware neural networking. Recently, Y. Shi et al. [144] developed a vdW heterostructure RRAM device based on multi-layered hexagonal boron nitride (h-BN) as the synapse medium. The device had a configuration of metal/h-BN/metal (see Figure 6j–l) with various metals like Ag, Au, Ti, and Cu being used. Depending upon the nature of the pulse voltage and the choice of electrode material, both volatile and non-volatile switching were achieved in the device. In the volatile switching regime, the device exhibited a very low power consumption of 0.1 fW in the standby mode and 600 pW for the SET transition, both of which are highly suited for the commercial realization of these devices. Further, the device was capable of mimicking several synapse functionalities like PPF, PPD, and STDP. The underlying mechanism for the device performance was identified as a combination of both conductive bridge formation due to the metal ions and the boron vacancy migration from the h-BN to the anode. Similarly, R. Xu et al. [150] fabricated a vertically stacked vdW heterostructure device composed of two MoS_2_ monolayers sandwiched between a top Cu electrode and bottom Au electrode. The device works on the basis of Cu ion diffusion through the atomically thin MoS_2_ layers, thereby lowering the switching voltage to around 0.1∼0.2 V. The device also exhibits synaptic learning rules like STDP responses with high consistency. Such a low-power synaptic device is ideal for practical neuromorphic computing applications. A vertical MoS_2_/graphene van der Waals heterojunction [151] was also used for emulating LIF neuron response with properties like threshold firing and neuron refractory period.

## 5. Emerging Neuromorphic Applications

In the previous section, we discussed several RRAM platforms with diverse material compositions capable of emulating neuronal behaviors. In recent years, the field of neuromorphic computing has found novel applications in emerging applications like the emulation of novel biological responses, computer vision, advanced computing architectures, audio & speech processing, medical applications, sensors, etc. Table 3 summarizes some of the recent neuromorphic applications implemented using RRAM devices.

**Figure 7:**
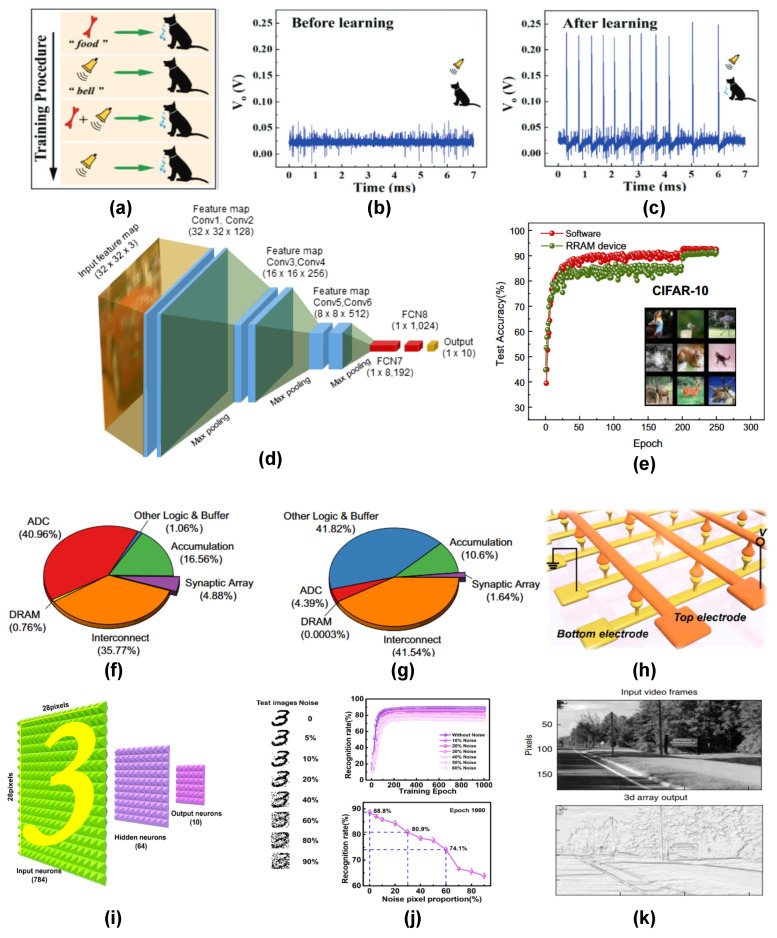
(**a**) Schematic of the pavlov’s dog associative learning implemented using a reconfigurable NbO_*x*_ memristor device. Electrical voltage output (**b**) before the training process & (**c**) after the training process. Reprinted with permission under a Creative Commons CCBY License from [161]. (**d**) Schematic of the VGG8 neural network architecture used for CIFAR-10 pattern recognition using a GeO_*x*_-based RRAM cell. (**e**) CIFAR-10 image recognition accuracy as a function of the training epochs for the GeO_*x*_-based RRAM cell in comparison with the purely software-based training. Inset shows sample images of the CIFAR-10 dataset. Pie charts showing the system level (**f**) energy & (**g**) latency distribution of the various hardware peripherals used for the image recognition simulation. Reprinted with permission under a Creative Commons CCBY License from [154]. (**h**) Schematic of the stacked 3D memristor array composed of Pt/HfAlO_*x*_/TaN devices. (**i**) Illustration of the fully connected artificial neural network used for MNIST data pattern recognition. (**j**) MNIST pattern recognition rate (%) as a function of the number of training epochs and the amount of noise (%) added to input image. Reprinted with permission from [16]. Copyright © 2024 American Chemical Society. (**k**) Comparison of software & hardware generated edge detection from input video frames based on a 3D integrated Pt/HfO_2_/Ta memirstor array. Reprinted with permission from [156]. Copyright © 2024 Springer Nature.

Emulation of biological learning behaviors using RRAM devices has been one of the earliest research attempts in neuromorphic research. Apart from synapse and neuron realization, several advanced learning behaviors were also demonstrated using RRAM devices. Associative learning is a biological learning behavior that works by linking the correlation between separate events or stimuli so that they form a connection inside the brain. It has been extensively studied in neuromorphic computing systems, mainly due to their correlated learning attributes. Recently, Y. Pei et al. [153] demonstrated Pavlovian associative learning functions using a carbon-quantum-dot-based memristive device. TEM images revealed that carbon filaments developed inside the device were responsible for the conduction mechanism. In order to demonstrate the Pavlovian learning rule, the device was subjected to stimuli corresponding to a bell and food in a real-life situation. Initially, the device was in the HRS state when the bell stimulus was fed, which switched to LRS when the food stimulus was given. After the combined application of both stimuli, the system responds to the bell stimulus alone afterwards, which indicates the associative learning behavior of the device. The device was also found to exhibit digit recognition with a high accuracy of more than 90%. Classical Pavlov learning was demonstrated using spike based response signals (see Figure 7a–c) by C.Y. Han et al. [161]. They used a reconfigurable memristor device based on NbO_*x*_ switching layer as both the synapse and neuron to trigger a threshold spiking behavior, as shown in Figure 7b,c. Pavlovian learning has also been demonstrated in several recent RRAM-based reports [162,163]. Pattern recognition is also another advanced learning behavior of the brain that enables the recognition and distinction of patterns with repeated training. Image recognition based on handwritten digit datasets has been reported using RRAM devices in several recent reports with a high degree of accuracy [163,164]. K. Udaya Mohanan et al. [154] reported the inference accuracy of a GeO_*x*_-based RRAM device on a simulated convolutional neural network (CNN) architecture using the CIFAR-10 dataset (see Figure 7d–g). They reported a pattern recognition accuracy of 91.27% using a system-level simulation incorporating a fully neuromorphic architecture. Image recognition based on 3D flexible crossbar memristor arrays based on Pt/HfAlO_*x*_/TaN configuration was recently reported by T.Y. Wang et al. [16] (see Figure 7h). With a very low-energy consumption of 4.28 aJ/spike, the device could recognize standard database images with additional noise pixels with a high level of accuracy. K. Wang et al. [165] also reported a low power 2D MoS_2_ based memristor device with a pattern recognition accuracy of 90.37%.

Another important application is the sparse coding of input data, which allows for a minimal representation of the input data, thereby developing a dictionary that helps in identifying patterns in a future dataset. P. M. Sheridan et al. [166] reported a 32×32 crossbar memristor array based on tungsten oxide (WO_*x*_) capable of sparse representation of an input image into a dictionary of 20 elements. The device was found to be capable of reconstructing more complex input images with the dictionary elements already recorded. Recently, D. H. Lim et al. [167] reported a 1 Gb PCM-based memristor array with a 39 nm process technology. By studying the statistical parameters involved in resistance drift, a neural network was designed to demonstrate a spontaneous sparse learning scheme. P. Lin et al. [156] reported a 3D memristive circuit composed of 8 layers capable of both pattern recognition and edge detection in videos (see Figure 7k). In spite of the device-to-device variations, these 3D array circuits were capable of performance on par with purely software implementations.

**Figure 8:**
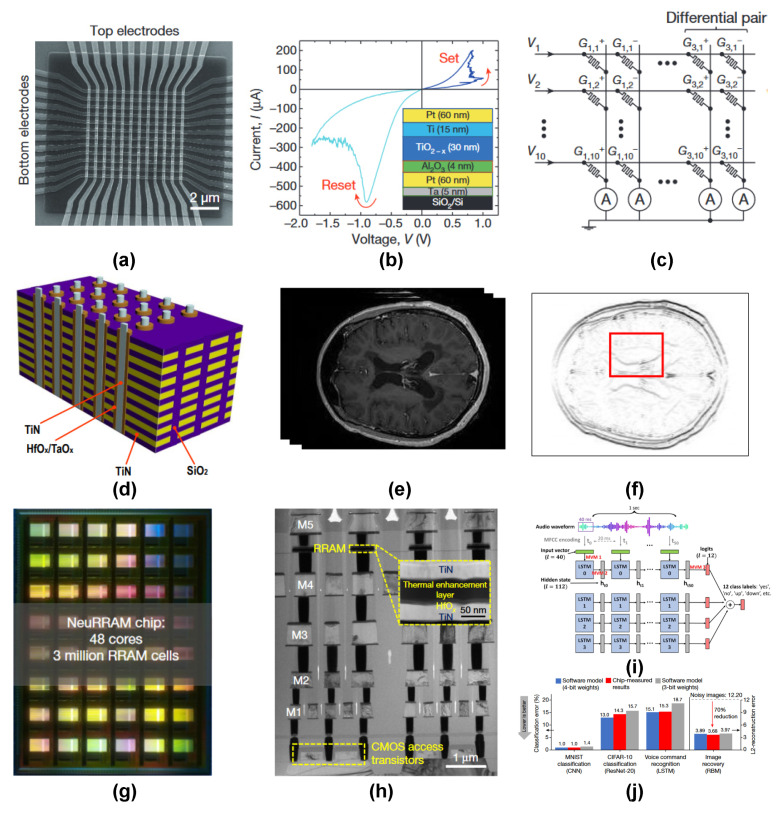
(**a**) SEM image of 12×12 crossbar array composed of Al_2_O_3_/TiO_2_ memristor devices. (**b**) Analog switching characteristics of the memristor device. Inset shows the device schematic. (**c**) Schematic of a crossbar array implementing a 10×6 fragment of a single-layer perceptron neural network. Reprinted with permission from [168]. Copyright © 2024 Springer Nature. (**d**) Device structure of a 3D VRRAM architecture composed of TiN/HfO_*x*_/TaOx/TiN memristor devices. (**e**) Specimen MRI image of the brain as the input data. (**f**) VRRAM based edge detection using 3D Prewitt kernels for convolving the brain MRI image. Reprinted with permission under a Creative Commons CCBY License from [169]. (**g**) Micrograph image of the NeurRRAM chip. (**h**) Cross-sectional TEM image showing the different layers inside the VRRAM stack. (**i**) Illustration of the LSTM model used for Google speech command recognition. (**j**) Bar plot showing the classification error (%) for various dataset/model combinations. Side panel shows the noisy image recovery error for the RBM model. Reprinted with permission under a Creative Commons CCBY License from [17].

Lately, several RRAM-based chips have been reported for implementing deep neural networks with minimal energy efficiency as compared to conventional CMOS chip designs. The earliest demonstration of a fully on-chip trainable RRAM chip was reported in 2015 by M. Prezioso et al. [168] using a binary oxide Al_2_O_3_/TiO_2−*x*_ switching stack. They implemented the on-chip training of a 12×12 RRAM crossbar array (see Figure 8a,b) for the classification of a 3×3 binary image using a modified delta training algorithm. They also implemented a novel weight mapping scheme using differential conductance pairs of memristors having opposite charge polarity (see Figure 8c). A 3D vertical integration-based RRAM (VRRAM) chip was reported by Q. Huo et al. [169] for brain MRI edge detection (see Figure 8d–f) with a high energy efficiency of 8.32 tera-operations per second per watt (TOPS/W).

In 2023, IBM reported a PCM-based neural inference chip “HERMES” [170], which was based on the compute-in-memory architecture. The HERMES chip used a staggering ∼16 million PCM devices and achieved a high level of parallelism with a throughput of 63.1 TOPS and an energy efficiency of 9.76 TOPS/W. The compute capabilities of the chip were evidenced by the high level of performance in generating text-based captions on the Flickr8k dataset using a long short-term memory network (LSTM). The biggest performance gain reported among RRAM-based deep learning chips (at the time of publication) has been reported by the “NeuRRAM” chip [17] (see Figure 8g). The chip has 48 synaptic cores composed of 65,536 RRAM devices and 256 CMOS-based neuron circuits. The NeuRRAM chip is fully reconfigurable and can support both training and inference with full data & model parallelism capabilities. NeuRRAM consumes 2.3× less energy as compared to its digital counterparts and records comparable performance across several deep learning models. On the Google speech command recognition task, the NeuRRAM chip recorded an impressive performance of 84.7% using an LSTM network.

## 6. Challenges & Future Outlook

RRAM devices have been extensively used for neuromorphic computing applications over the past decade. However, several issues still remain unsolved, both at the device level and at the array level. The stochastic nature of conductive filament growth and the lack of a clear understanding of the conduction dynamics are the primary hindrances to the future growth of RRAM device research and its eventual commercialization. In order for RRAM devices to be practically useful for neuromorphic computing applications, device non-ideality [171] issues need to be addressed carefully. These include a variety of issues like cycle-to-cycle conductance variations, device-to-device variations, conductance drift, high ON current, etc. Both the device-to-device variability and the cycle-to-cycle variability can be minimized with standardized device fabrication strategies, which have been reported in several of the reports discussed here. Further optimizations have to be focused both at the device architectural level and at the material selection choices to minimise issues like the high ON current observed in RRAM devices. Device architectural innovations like multi-terminal device design [172], 3D stacking [173], interface & filament modulation [174], defect engineering [175], etc. have been effective in mitigating RRAM device-level non-idealities. For neuromorphic emulation, the device level conductance update linearity and symmetry have also proven very critical [176,177]. From a materials perspective, new & innovative material choices need to be identified with inherent properties adaptable to neuromorphic emulation. Although traditional metal oxide layers are still the predominant choice in neuromorphic research, emerging material choices like 2D materials and vdW heterostructures can be a suitable alternative given their scope for dimensional scaling and energy efficiency. At the crossbar array level, several issues like IR drop, high read-out currents, and large peripheral circuits need to be addressed [178]. Another key issue is the algorithm-level innovations required for overcoming or minimizing the effect of device-level imperfections. Apart from attempts at compressing neural network architectures [179], RRAM weight mapping algorithms [180], noise-aware training algorithm [181,182] and fault mitigation algorithms [183] have been reported with much success in recent literature. An alternative strategy is the hardware-software codesign paradigm, where the inherent stochasticity of these devices is incorporated into neural network training and/or inference algorithms [184,185]. Finally, the technological adaptation of RRAM devices for neuromorphic computing requires major innovations in terms of scaling capabilities. As the demand for computing power increases, so should the scalability of RRAM-based crossbar array architectures. Novel architectural solutions like horizontal stacked 3D structure & vertical 3D integration [18] can be possible candidate solutions for addressing scaling issues. Finally, anomalies in the analysis & reporting of device performance still exists, and detailed reports have been recently published on strategies to mitigate such issues [186,187,188]. In conclusion, with further research focused on the device-, circuit-, and algorithm-level architectures, RRAM devices can truly attain their full potential in realizing practical neuromorphic-based computing systems. 

## Figures and Tables

**Table 3:** Summary of emerging neuromorphic applications implemented using RRAM devices.

Neuromorphic Applications	Device Composition	Highlights	Ref.
**Biological Emulation:**			
(a) Synapse	Ti/Ta/HfO_2_/Al_2_O_3_/Pt	LTP/LTD	[85]
(b) Neuron	Au/Ti/VO_2_/Al_2_O_3_/Au	LIF	[87]
(c) STDP	W/Al/PCMO/Pt	Asymmetric STDP	[152]
(d) Metaplasticity	Pt/WO_3_/Pt	Metaplasticity effects on STDP	[88]
(e) Heteroplasticity	Pt/TiO_2−*x*_/Pt	Gated modulation of plasticity	[91]
(f) Associative learning	Pd/C−QD/Ga_2_O_3_/Pt	Pavlovian learning	[153]
**Computer Vision:**			
(a) Image classification	Ni/GeO_*x*_/p^+^Si	91.27% accuracy on CIFAR10/VGG8	[154]
(b) Image segmentation	TiN/Ta/TaO_*x*_/TaN	97% accuracy on DRIVE/U-Net	[155]
(c) Video edge detection	Pt/HfO_2_/Ta	3D RRAM circuit	[156]
**Temporal &** **Audio Processing:**			
(a) Time series prediction	Pt/HfO_2_/TiN	0.04% error rate on Mackey-Glass time series data	[157]
(b) Spoken digit classification	Ti/TiO_*x*_/TaO_*y*_/Pt	99.6% accuracy on NIST TI-46 database	[158]
(c) Speech recognition	TiN/TaO_*x*_/HfO_*x*_/TiN	84.7% accuracy on Google speech command/LSTM	[17]
**Natural Language** **Processing:**			
(a) Text generation	Pt/TiO_*x*_/Ti	Antimicrobial peptide (AMP) sequence generation	[159]
**Other Applications:**			
(a) Medical Diagnosis	Pt/HfO_2_/TiN	80% accuracy in ADHD analysis	[157]
(b) Security Application	Cu/HfO_2−*x*_/*p*^++^Si	Physically unclonable function	[160]

## Data Availability

Data are contained within the article.
